# Aligning Computer Vision with Expert Assessment: An Adaptive Hybrid Framework for Real-Time Fatigue Assessment in Smart Manufacturing

**DOI:** 10.3390/s26020378

**Published:** 2026-01-07

**Authors:** Fan Zhang, Ziqian Yang, Jiachuan Ning, Zhihui Wu

**Affiliations:** 1 College of Furnishings and Industrial Design, Nanjing Forestry University, Nanjing 210037, China; zfan1003@njfu.edu.cn (F.Z.); wzh550@sina.com (Z.W.); 2 Qingdao Grace Chain Software Ltd., Qingdao 266071, China; ningjiachuan@guanchengsoft.com; 3 Jiangsu Co-Innovation Center of Efficient Processing and Utilization of Forest Resources, Nanjing 210037, China

**Keywords:** work-related musculoskeletal disorders, CNN, LSTM, ergonomic assessment, posture recognition

## Abstract

To address the high incidence of work-related musculoskeletal disorders (WMSDs) at manual edge-banding workstations in furniture factories, and in an effort to tackle the existing research challenges of poor cumulative risk quantification and inconsistent evaluations, this paper proposes a three-stage system for continuous, automated, non-invasive WMSD risk monitoring. First, MediaPipe 0.10.11 is used to extract 33 key joint coordinates, compute seven types of joint angles, and resolve missing joint data, ensuring biomechanical data integrity for subsequent analysis. Second, joint angles are converted into graded parameters via RULA, REBA, and OWAS criteria, enabling automatic calculation of posture risk scores and grades. Third, an Adaptive Pooling Convolutional Neural Network (CNN) and Long Short-Term Memory Network (LSTM) dual-branch hybrid model based on the Efficient Channel Attention (ECA) mechanism is built, which takes nine-dimensional features as the input to predict expert-rated fatigue states. For validation, 32 experienced female workers performed manual edge-banding tasks, with smartphones capturing videos of the eight work steps to ensure authentic and representative data. The results show the following findings: (1) system ratings strongly correlate with expert evaluations, verifying its validity for posture risk assessment; (2) the hybrid model successfully captures the complex mapping of expert-derived fatigue patterns, outperforming standalone CNN and LSTM models in fatigue prediction—by integrating CNN-based spatial feature extraction and LSTM-based temporal analysis—and accurately maps fatigue indexes while generating intervention recommendations. This study addresses the limitations of traditional manual evaluations (e.g., subjectivity, poor temporal resolution, and inability to capture cumulative risk), providing an engineered solution for WMSD prevention at these workstations and serving as a technical reference for occupational health management in labor-intensive industries.

## 1. Introduction

Among musculoskeletal disorders (MSDs), work-related musculoskeletal disorders (WMSDs) are now a central concern in global occupational health, largely because they are closely linked to work activities and environments [1]. In fact, the International Labour Organization (ILO) officially recognized WMSDs as occupational diseases as early as 2002 [2]. Epidemiological data indicate that the overall prevalence of WMSDs among Chinese workers reaches 41.2% [1], with high incidence rates in the neck, shoulders, and lower back. Women are at 1.5 times higher risk than men, especially in labor-intensive sectors such as construction, manufacturing, and agriculture. Furniture manufacturing, a typical labor-intensive industry, presents particularly high risks for WMSDs. This is especially true at manual edge-banding workstations, where tasks often involve prolonged physical effort, repetitive motions, and sustained awkward postures. Existing research [3,4,5] reports that WMSDs affect between 26.60% and 53.90% of workers in this industry, with the shoulders, hands, wrists, and lower back being the most vulnerable regions. Targeted risk assessment solutions are urgently needed [6]. Therefore, regular ergonomic musculoskeletal disorder assessments can play a crucial role in monitoring worker health within occupational safety management [7].

In furniture manufacturing, three approaches are commonly used to assess whether a worker’s posture is ergonomic: observation, direct measurement, and machine vision. When evaluating the postures of operators who apply edge banding by hand, the most frequently adopted observation tools are the Rapid Entire Body Assessment (REBA) [8], the Rapid Upper Limb Assessment (RULA) [9], and the Ovako Working Posture Analyzing System (OWAS) [10]. These tools divide the body into several segments and convert postures into simple scores based on how tiring each position is [11,12]. In practice, assessments are often carried out by human experts through observation. However, manual checks are prone to inconsistency. The same person may rate the same posture differently at different times, and different assessors may disagree with one another. These variations reduce the repeatability of the evaluation standards [13].

Direct measurements use invasive tools. These include electromyography (EMG) [14], force plates, and inertial sensors [15,16,17,18]. They collect objective data such as muscle movement and joint pressure. However, sensors need to be attached to the human body. This may obstruct precise work [19]. In contrast, machine-vision-based assessment collects data using visual equipment. This approach can automatically identify physical features associated with fatigue [20,21,22], enabling real-time and automated risk analysis. As a result, it has become a preferred option for deployment in industrial settings [23,24].

This paper focuses on visual-technology-based methods for assessing WMSDs. Compared to direct measurement, visual techniques provide a non-invasive approach for evaluating posture-related risks. They offer objective and consistent postural assessment while minimizing interference with the worker’s natural motions [25].

Vision-based assessments of WMSDs center on deep-learning-driven human pose estimation. Pose estimation algorithms recognize human body joints and the angles of these joints. The data they provide form the basis for ergonomic assessments [26]. DeepPose [27] first used CNNs for end-to-end joint coordinate regression. It surpassed the limits of features set by hand. OpenPose [28] can estimate the poses of many people at the same time. It does this with joint heatmaps and Part Affinity Fields (PAFs). But in manual edge-banding workstations with lots of equipment, joint mismatches take place quite often. HRNet [29] keeps high-resolution images during the whole process. It makes joint localization more accurate for small-sized human bodies. Still, this model has a large number of parameters. Ereena et al. employed temporal modeling of visual features [30], feeding MediaPipe-extracted 2D joint coordinates into an LSTM to output action categories, capturing dynamic pose evolution. Considering objective constraints such as the manual edge-banding workstation environment and hardware deployment costs, this paper constructs pseudo-3D poses using MediaPipe-output 2D coordinates and z-axis depth information, balancing accuracy with practicality.

To predict the ultimate fatigue index of workers’ operational postures, we apply deep learning to overcome the subjective limitations of traditional assessments [31]. Previous research has demonstrated that CNNs possess strong local feature extraction capabilities and have dominated the field for decades [32,33,34,35]. However, risk fatigue is a time-accumulated outcome, and standalone CNNs cannot capture cumulative risk; they require integration with temporal models. Yan et al.’s Spatio-Temporal Graph Convolutional Network (ST-GCN) [36] pioneered the integration of spatial relationships within skeletal sequences with temporal dependencies. Li et al.’s MotionMixer model achieves long-term 3D pose prediction using a pure MLP architecture [37]. Long Short-Term Memory (LSTM) [38] networks can be used to address the vanishing gradient problem in traditional RNNs through gating mechanisms (forgetting gate, input gate, output gate), making them suitable for processing multi-frame temporal data [39]. The effectiveness of CNN–LSTM fusion models in human recognition has been demonstrated. Zhang et al. [40] employs CNN and LSTM units as backbone networks to extract key point information and temporal information from sports video frames, respectively. Mengjiao Zhao et al. [41] utilizes MediaPipe and CNN to extract features from acquired human key points, combining them with LSTM algorithms to recognize Pilates poses in real-time videos. Incorporating LSTM modules into CNN networks compensates for CNN’s limitations in modeling long-term temporal sequences.

From the groundbreaking design of AlexNet [42] to ResNet’s [43] innovative use of residual connections to solve the problem of degradation in very deep networks, the development of CNN architectures has continually focused on improving their ability to represent features [44,45,46]. Traditional CNNs treat all channels and spatial locations the same way when extracting features. They cannot easily focus on key features related to the task. As a result, these models pay too much attention to unnecessary information. At the same time, they fail to capture important details well. Attention mechanisms have become a key technology to improve CNN performance [47,48]. Channel attention has become a popular research topic. It has a simple structure. It can be easily added to existing CNN frameworks. It can also catch the links between feature channels accurately. However, most current methods have a problem. They need to balance better performance and higher computing costs. SENet [49] created the basic channel attention design. Modules such as CBAM and DA made performance better. They used multidimensional combinations [50,51]. But none of these methods solved two main issues. They did not fix the accuracy drop from dimension reduction. They also failed to handle efficiency problems from global modeling. The Efficient Channel Attention (ECA) [52] mechanism solves this balance problem. It uses three new design ideas: no dimension reduction, local cross-channel connections, and flexible kernel size choice. It achieves a solution that is both light and high-performing. The ECA module [53] works well. It helps CNN models to improve in picking out key features from sEMG signals. Feng et al. [54] combines ConvMixer with the ECA module. This combination can correctly diagnose ADHD by using EEG signals.

This study integrates the ECA mechanism [52] into the target model architecture. While CNNs are capable of extracting local patterns from feature sequences and identifying high-risk indicators in short timeframes, the added ECA module employs efficient 1D convolution to compute channel attention. This allows the network to focus more effectively on locally relevant features critical for fatigue detection. Worker fatigue accumulates gradually over time; we incorporate LSTMs [38], which effectively model time series relationships, and address a key practical issue: fatigue indexes can increase even when posture risk scores remain constant.

With its dual-branch architecture and ECA attention mechanism, the proposed model is capable of representing the complex, nonlinear dynamics of fatigue development. Enhanced by comprehensive input features and ECA, the model demonstrates robust performance within the targeted manual edge-banding scenario, showing potential for adaptation to similar repetitive industrial tasks. Additionally, we employ deep learning techniques based on RULA [9], REBA [8], and OWAS [10] criteria for ergonomic assessment. By calculating human joint angles from collected field video data, we derive full-body posture scores and risk levels to achieve an accurate evaluation and prediction of ergonomic hazards. This enables real-time analysis and forecasting of musculoskeletal disorder risks for manual edge-banding workers in furniture manufacturing plants. Most existing deep learning approaches merely perform regression on static RULA/REBA scores. However, this “learning from rules” approach inherits the limitations of static formulas, failing to capture the physiological reality of cumulative fatigue that characterizes comprehensive expert assessments.

The main contributions of this paper are as follows:

(1) A dual-drive feature fusion framework: Unlike prior works that rely solely on black-box deep learning for feature extraction (e.g., [30]) or static rule-based scoring (e.g., [55]), this paper proposes a formally articulated “dual-drive” architecture. By mathematically embedding expert rules (RULA/REBA/OWAS scores) into the feature space of a deep neural network, we bridge the gap between biomechanical interpretability and data-driven nonlinearity. This hybrid approach allows the model to capture fatigue accumulation that static formulas miss, while maintaining ergonomic validity that pure-data-driven models often lack.

(2) Context-aware adaptive pooling strategy: Addressing the limitation of standard pooling in capturing variable-speed industrial motions, we introduce a quantifiable adaptive strategy based on the temporal window threshold (T = 7). This mechanism formally differentiates between “Salient Feature Extraction” for sustained postures and “Lossless Feature Expansion” for rapid movements. This structural innovation prevents information loss from high-frequency risk signals, which is common in general-purpose networks (e.g., YOLO-based approaches [56]), ensuring sensitivity to transient high-risk behaviors.

## 2. Related Work

### 2.1. Traditional Ergonomic Assessment Methods

To quantitatively assess WMSDs, ergonomics experts typically rely on two main types of traditional assessment methods: self-report and observation-based tools.

Self-report methods depend largely on workers’ personal feedback. The Nordic Musculoskeletal Questionnaire (NMQ) [57] is the most commonly used tool. This questionnaire focuses on pain conditions in nine body parts. These parts include the head, neck, and shoulders. It records how frequent and severe pain has been in the past 12 months. It works well for large-scale group screening. But this method has a drawback: it cannot collect objective physiological or biomechanical data, such as joint angles and force levels.

Observation-based assessments are the most popular method in industrial settings. Globally recognized tools like RULA, REBA, and OWAS are favored due to their comprehensive coverage of risk factors and practical ease of use [11,12]. To address WMSDs, an increasing number of scholars have adopted integrated approaches that combine one or more traditional assessment methods with machine vision technology.

#### 2.1.1. RULA Evaluation Method

**Figure 1:**
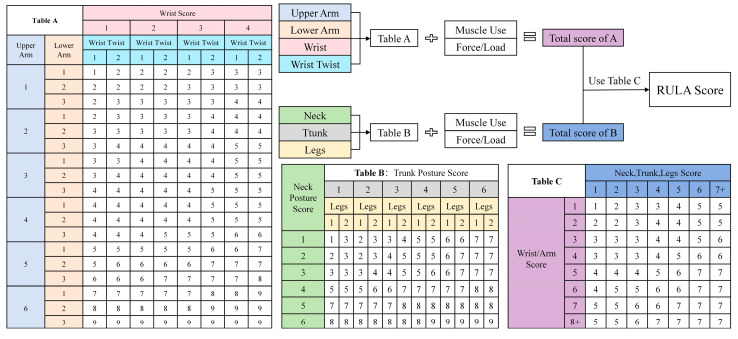
RULA rating sheet.

RULA [9,58], developed by researchers at the University of Nottingham, UK, focuses on the postural load assessment of the upper body (including upper arms, lower arms, wrists, neck, shoulders, and back). It calculates load scores by integrating ratings of two body groups: Group A (upper arms, lower arms, wrists) and Group B (neck, trunk, legs). The assessment results are converted into risk scores ranging from 1 to 7, corresponding to four risk levels. RULA is particularly suitable for industrial scenarios involving repetitive manual operations. The specific process is illustrated in Figure 1. However, RULA inadequately covers lower limb postures and cannot fully reflect the whole-body load associated with manual edge banding tasks involving “prolonged standing and bending.”

#### 2.1.2. REBA Evaluation Method

**Figure 2:**
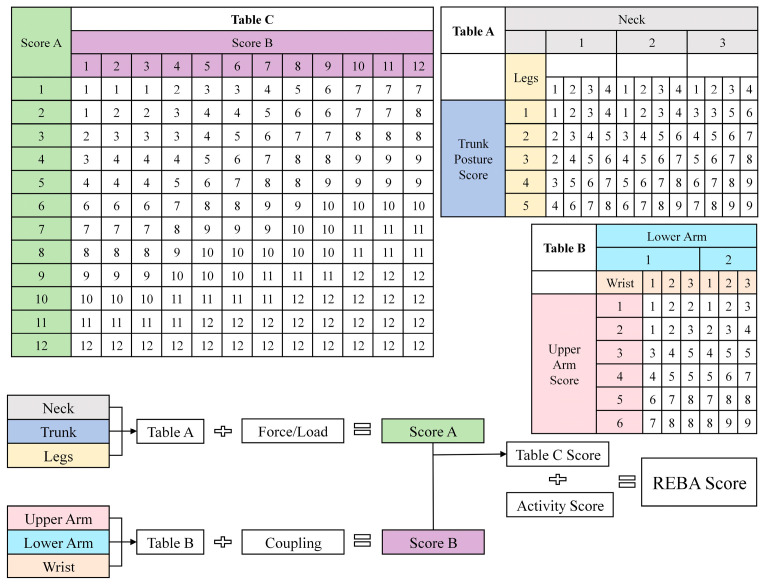
REBA rating scale.

REBA [8,58,59], a core ergonomic musculoskeletal risk assessment tool, is designed to comprehensively evaluate the impact of work postures on the human musculoskeletal system. REBA is built as an extended form of RULA. It is designed to assess postural strain across the entire body, covering major body segments such as the arms, trunk, and legs. This feature makes it fit for many work scenarios that require full-body coordination—including healthcare, manufacturing, and logistics. The method follows steps similar to those of RULA, with detailed assessment procedures shown in Figure 2.

#### 2.1.3. OWAS Evaluation Method

**Figure 3:**
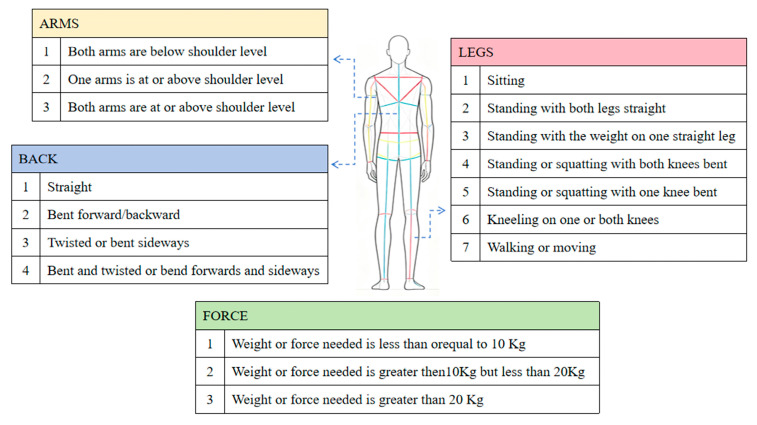
OWAS rating scale.

The OWAS [10,60] was developed by Finland’s Ovako company (headquartered in Helsinki, Finland). It integrates 4 back postures, 3 arm postures, 7 lower limb postures, and 3 load categories into 252 combinations. These are classified into 4 risk levels via a four-digit code to determine the exposure hazard level of a worker’s posture, as shown in Figure 3. We convert the joint angles and key point coordinates extracted by MediaPipe into the four core dimensions required for OWAS assessment: “back, arm, leg, and load.” Workers at manual edge-banding stations only lift heated irons (load < 10 kg), so the load level is set to 1. We define the OWAS codes “AC1–AC4” as “1–4”, respectively. Predefined code mappings are used to calculate posture scores (OWAS score) and risk categories (OWAS risk, levels 1–4), which are stored for follow-up analysis.

This system is based on Python 3.8 and pose recognition tools. It studies the key body points and joint angles of human beings. It gives scores to the upper limbs and torso areas. The scoring follows fixed rules. This approach automates the scoring process. The system supports three widely used posture risk assessment methods: RULA, REBA, and OWAS. In practice, we convert raw joint angle data into quantifiable metrics.

### 2.2. Ergonomic Evaluation Based on Human Posture Recognition

Two types of posture-recognition methods have been developed. They are direct sensor measurement and indirect extraction through machine vision. These methods have reduced the subjective bias in traditional WMSD assessment. Direct measurement methods rely on wearable sensors [61]. Souha et al. [62] designed an upper-body WMSD assessment system based on an inertial measurement units (IMUs) technology for the cable manufacturing industry. The team optimized sensor positions and angle calculation programs. The system showed results that were consistent with those obtained using traditional methods. Troy et al. [63] used surface electromyography (Semg) and electrogoniometry (EG) tools. They recorded physical workloads of sawmill workers during repetitive tasks. This method made it possible to show workloads visually and give feedback. Andrew et al. [64] used wearable sensors to assess surgeons’ postures and provide real-time feedback to help correct unhealthy positions. These direct measurement methods can collect highly accurate data. But they have big drawbacks in manual edge-banding workstations. IMU sensors need to be fixed on wrists and waists. They might get in the way of precise work movements. sEMG electrodes must touch the skin directly. They may fall off during long work shifts. Additionally, the high cost of this kind of equipment typically stops it from being used widely in factories.

Indirect extraction methods rely on machine vision. Their main advantage is non-invasiveness. They have become the first choice for WMSD assessment in factories [25,26]. These methods use RGB cameras to record video. They combine the video data with pose estimation algorithms. The combined process extracts joint coordinates. This way, it does not disturb the normal work on the site.

Kiraz et al. [65] applied deep learning to find human joint positions from video in real time. The results provided basic data for posture assessment. Similarly, Hossain et al. [55] obtained 3D human key points from RGB images. These points were used as inputs for an REBA-based risk assessment to predict musculoskeletal risk levels automatically. Spectre [66] Model 72 combined wireless motion trackers with CNNs. It realized automatic work posture classification. It also finished automatic RULA scoring. Srimantha et al. [67] trained machine learning models, including Support Vector Machines (SVMs) and decision trees. The training used sEMG data and achieved high accuracy in predicting risk during material handling tasks.

These studies prove an important point. Machine vision methods surpass the subjective limits of traditional assessments. They also fit well with manual edge-banding workstations because of their non-invasive feature. However, dynamic risk buildup needs special handling. A typical case is fatigue from repeated bending movements. This process requires modeling with time-related feature data. The finding provides the conceptual basis for this paper. The paper combines posture recognition (spatial features) and deep learning temporal models (temporal features).

## 3. Method

### 3.1. System Architecture

This study focuses on workers at manual edge-banding stations in furniture factories. They do dynamic tasks while keeping fixed body positions. Bending is one common example of these positions. This mix of movement and static posture brings big risks of getting WMSDs.

**Figure 4:**
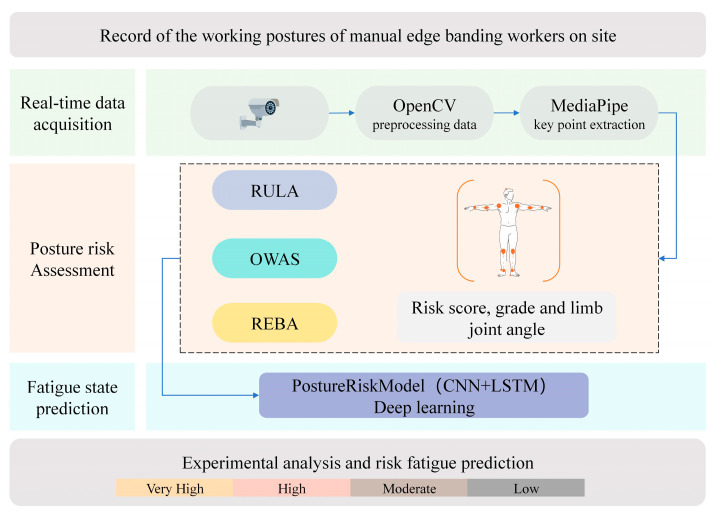
System architecture.

To enable continuous and intelligent monitoring of these risks, we developed a real-time fatigue prediction system based on video recordings of workers’ postures. First, it applies OpenCV to analyze the videos. For each video frame, the program first extracts the pixel matrix, which is initially in BGR color format with dimensions H × W × 3. This matrix is then converted from BGR to RGB to align with the channel order expected by the MediaPipe framework. Next, the image resolution is adjusted to a fixed 640 × 480 pixels. Bilinear interpolation is used in this process. It lowers computing costs and ensures uniform input data. After that, the MediaPipe Pose model is used. It detects key body points from monocular RGB images. A total of 33 key joints are output. Each joint is linked to a certain body part. Normalized parameters are provided for every joint. These parameters include 2D/3D coordinates (x, y, z) and a visibility score. MediaPipe z value stands for relative landmark depth. It does not show absolute metric depth. Therefore, joint angles are primarily calculated using 2D vectors, while the z-value assists in determining viewpoint, enabling a form of pseudo-3D pose estimation. Finally, posture risk scores—computed using the RULA, REBA, and OWAS assessment tools—are combined with the multidimensional posture features extracted above. This integrated dataset is passed into a deep learning model named PostureRiskModel, which is built on a CNN–LSTM architecture. The model outputs real-time fatigue indexes, supporting continuous monitoring of fatigue risk in practical settings. The overall system workflow is illustrated in Figure 4.

To avoid ambiguity, this paper uniformly uses the term “Fatigue Index” to represent the quantitative assessment of worker fatigue. Specifically, it refers to the continuous numerical value predicted by the model, which approximates the expert-scored Borg RPE scale (range: 6–20).

### 3.2. Limb Angle Calculation

MediaPipe is a lightweight, real-time computer vision framework developed by Google. It uses a two-step structure consisting of human detection (BlazeDetect) and key point tracking (BlazePose Landmarker) [68]. It finally outputs 3D joint coordinates. Its light design, high accuracy, and real-time ability meet the needs of industrial uses. It is tested on the COCO dataset and reaches an average accuracy rate of 78.9%. It works better than the traditional OpenPose method [28]. It also runs approximately three times faster than HRNet during inference [29].

**Figure 5:**
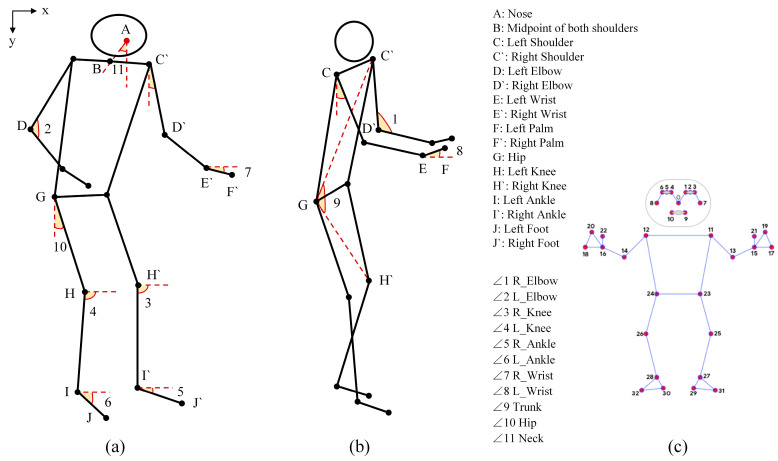
Relevant joint angles. (**a**,**b**) The angles calculated for the working posture. (**c**) MediaPipe’s 33 key points.

For musculoskeletal risk assessment using tools like RULA, REBA, and OWAS, the accurate calculation of joint angles is essential. A typical example is the shoulder flexion angle. So, the posture-recognition tool must provide reliable key joint coordinates. In this study, we selected over 20 core key points from MediaPipe’s full set of 33 landmarks, as shown in Figure 5. These points, such as the head, neck, and shoulders, are used for joint angle calculation and viewpoint determination.

Joint angles are computed using vector geometry. Angles are found by creating vectors from three connected key points. For example, the right elbow angle comes from three sets of coordinates. These coordinates belong to the right shoulder, right elbow, and right wrist. Let these three points be marked as R_Shoulder($x_{1},$ $y_{1}$), R_Elbow($x_{2}$, $y_{2}$), and R_Wrist($x_{3}$, $y_{3}$). Two vectors are created with the right elbow as the starting point. The inverse tangent function is used next. It calculates the angles between these vectors and the positive X-axis direction.

(1) $$\theta_{1}=atan2\left(x_{2}-x_{1},y_{2}-y_{1}\right)$$

(2) $$\theta_{2}=arctan2\left(x_{2}-x_{3},y_{2}-y_{3}\right)$$

The angle between two vectors is as follows:

(3) $$\theta_{right\_elboe}=\left|\theta_{2}-\theta_{1}\right|\times \frac{180^{{}^{\circ}}}{\pi }$$

The scoring criteria of the RULA and REBA methods assess upper limb and trunk posture relative to the vertical axis. So, this study converts the initially calculated joint angles into values measured with respect to the vertical Y-axis. Specifically, if an initial angle exceeds 90°, it is adjusted to 270°. For example, given an initial angle $\theta_{1}$, the adjusted angle $\theta_{1}^{\prime }$ is obtained using the following formula:

(4) $$\theta_{1}^{\prime }=\left|90^{{}^{\circ}}-\theta_{1}\right|$$

We calculate angles for seven key joints—elbow, knee, ankle, wrist, trunk, hip, and neck—using the vector-based method illustrated in Figure 5. This provides the basic needed to compute RULA, REBA, and OWAS posture risk scores. We fix the problem of missing joint data in individual frames by applying historical interpolation. Specifically, when a joint angle is absent in the current frame, we perform time series interpolation based on valid angle data from the five most recent consecutive frames. The interpolation formula used is as follows:

(5) $$\hat{\theta_{t}}=\frac{1}{n}\sum_{i=1}^{n}\theta_{t-i}\left(n\leq 5,\theta_{t-i}\neq NULL\right)$$

where $\hat{\theta_{t}}$ denotes the angle after frame interpolation for the current frame, and n represents the number of valid historical frames.

### 3.3. Fatigue State Prediction Based on CNN–LSTM

Worker fatigue stems from the long-term accumulation of risks associated with multi-frame work postures, thus requiring assessments to incorporate both single-frame spatial features and temporal characteristics of continuous postures.

While CNN–LSTM hybrid models have proven effectiveness in fields like drowsy driving, existing research mostly uses single-indicator inputs—lacking full integration of multidimensional risk metrics—and this results in constrained assessment accuracy. To address this limitation, this paper proposes a hybrid CNN–LSTM model based on the Efficient Channel Attention (ECA) mechanism for quantitatively assessing the fatigue index of workers’ operational postures. With nine-dimensional posture features as input (three types of raw risk scores, normalized extremal values, mean values, dynamic risk trends, temporal positions, perspective encoding, and data source encoding), the model comprehensively captures the essence of posture-related risks.

To ensure both biomechanical validity and robustness against environmental variations, these features were selected based on a three-tiered logic. (1) Expert Rules Priors: RULA, REBA, and OWAS scores are included to embed established ergonomic rules directly into the network, preventing the “black box” model from violating basic biomechanical principles. (2) Temporal Risk Aggregates: Features such as “Average Normalized Risk” and “Risk Increase Flag” are derived to explicitly encode the cumulative nature of fatigue, which cannot be captured by instantaneous joint coordinates. (3) Contextual Metadata: “Perspective Encoding” and “Data Source Type” are incorporated to calibrate the model against variations in camera angles and collection devices, ensuring the model learns generalized fatigue patterns rather than overfitting to specific viewpoints.

**Figure 6:**
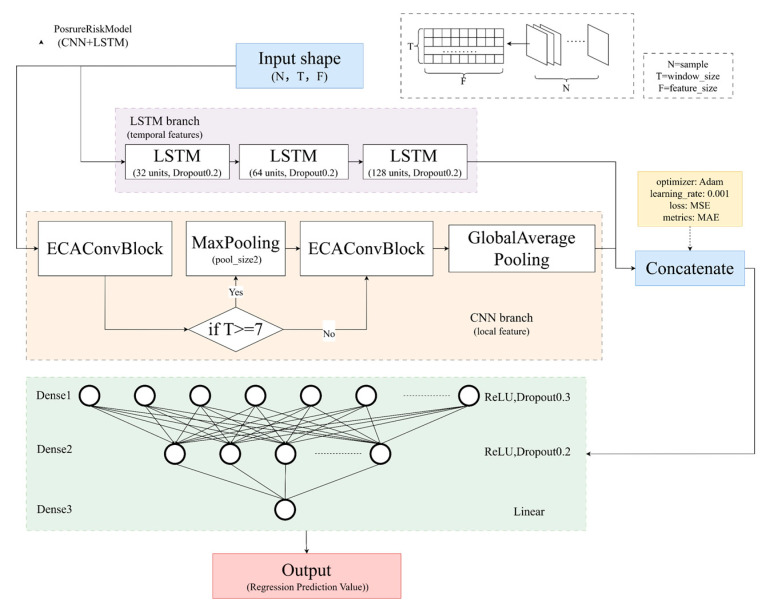
Model structure.

The model extracts local key information through a CNN branch equipped with an ECA attention mechanism, while the LSTM branch models long-term temporal dependencies. Features from the two branches are fused to enable the model to perform accurate regression prediction of fatigue indexes, as shown in Figure 6.

To enhance reproducibility, the definitions and derivation logic for the nine input features are summarized in Table 1.

#### 3.3.1. ECAConvBlock

**Figure 7:**
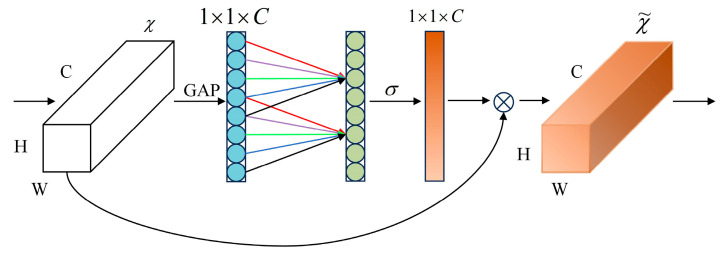
The structure of the ECA.

The ECA mechanism [52] is employed to adaptively recalibrate channel-wise feature responses without dimensionality reduction. It dynamically determines the kernel size, *k* based on channel dimension C (Figure 7) to capture cross-channel interactions:

(6) $$k={\left|\frac{{log}_{2}(C)}{\gamma }+\frac{b}{\gamma }\right|}_{odd}$$

where: γ = 2, b = 1, and C is the number of channels.

**Figure 8:**
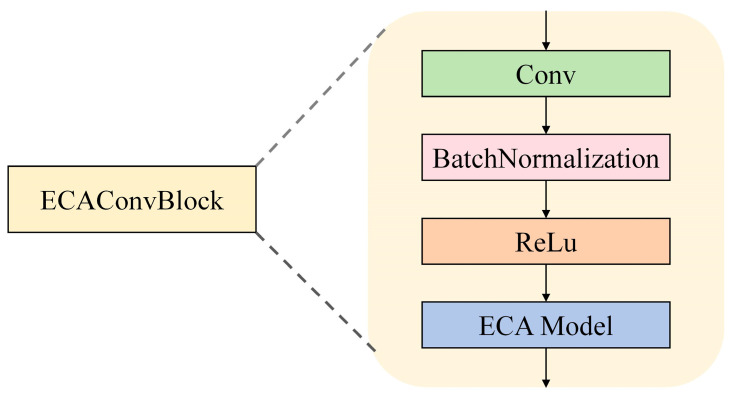
The structure of the ECAConvBlock.

For the CNN branch (Figure 8), the input is defined as the temporal feature matrix $X\in R^{N\times T\times F\;}$ (where F = 9).

We utilize 1D convolution to extract local correlations between features across adjacent time steps:

(7) $$Y_{cnn}=\sigma \left(\sum_{k=0}^{K-1}W_{k}\cdot X_{i+k}+b_{cnn}\right)$$

where k = 3, i.e., the convolution kernel size, meaning each kernel covers 3 consecutive time steps; $W_{k}\in R^{F}$ are the weights of the kth convolution kernel; $b_{cnn}$ is the bias term for the convolution operation; σ(⋅) = ReLU, i.e., the activation function introducing nonlinear features, defined as ReLU(x) = max(0,x).

To handle the varied action lengths in manual edge-banding tasks, we introduce a context-aware adaptive pooling strategy to prevent information loss typical in fixed pooling layers. The decision boundary T = 7 was determined through empirical optimization. Biomechanically, given the recording frame rate (30 fps), this duration (0.23 s) serves as a temporal filter to distinguish between transient involuntary tremors or sensor noise (short duration) and sustained voluntary postural adjustments (long duration). The strategy applies two distinct pathways:

(1) Salient feature extraction (T ≥ 7): for long action sequences (>0.23 s), max pooling is applied to retain peak risk signals (e.g., momentary extreme bending) while reducing temporal redundancy.

(2) Lossless feature expansion (T < 7): for short, rapid actions (<0.23 s), standard pooling is bypassed; instead, features are expanded via projection to turn limited temporal data into a higher-dimensional space, ensuring no key details are lost due to down-sampling.

Finally, global average pooling compresses the processed features into a stable vector for fusion.

#### 3.3.2. LSTM Module

The LSTM branch models the dynamic dependencies of the nine-dimensional risk features across T time steps. Through its gating mechanism (input, forget, and output gates), the network retains posture risk information over a 5–10 s window while dynamically updating memory with new frame data. Crucially, the forget gate mitigates the influence of occasional low-risk postures on fatigue prediction, thereby preventing misjudgments. The network adopts a three-layer architecture with dropout to reduce overfitting; the first two layers preserve sequential information, while the third integrates global window-based dependencies. The core calculations for time step t are presented here.

The forget gate determines whether the historical cell state should be forgotten, with an output range of [0, 1] (1 indicates fully retaining the historical state, 0 indicates fully forgetting the historical state). The formula is as follows:

(8) $$f_{t}=\sigma \left(W_{f}\cdot [h_{t-1},X_{lstm}(t)]+b_{f}\right)$$

The input gate determines the extent to which the current input updates the cell’s state, calculated by the following formula:

(9) $$i_{t}=\sigma \left(W_{i}\cdot [h_{t-1},X_{lstm}(t)]+b_{i}\right)$$

The candidate cell state generates the candidate update value for the current input feature, ranging from −1 to 1, calculated using the following formula:

(10) $${\tilde{c}}_{t}=tanh\left(W_{c}\cdot [h_{t-1},X_{lstm}(t)]+b_{c}\right)$$

The cell state update combines the historical state with the candidate update value from the current input, calculated using the following formula:

(11) $$c_{t}=f_{t}⊙c_{t-1}+i_{t}⊙{\tilde{c}}_{t}$$

The output gate determines the extent to which a cell’s state contributes to the hidden state, calculated by the following formula:

(12) $$o_{t}=\sigma \left(W_{o}\cdot [h_{t-1},X_{lstm}(t)]+b_{o}\right)$$

The hidden state is the output of the LSTM branch, carrying the temporal information of the current time step, as defined by the following formula:

(13) $$h_{t}=o_{t}⊙tanh(c_{t})$$

The parameters in the above formula are defined as follows: $h_{t-1}\in R^{H}$—hidden state at time step t−1; $X_{lstm}(t)\in R^{F}$—input features at time step t; $[h_{t-1},X_{lstm}(t)]\in R^{H+F}$—concatenation of hidden state and input; $W_{f},W_{i},W_{c},W_{o}$—gating weight matrices; $b_{f},b_{i},b_{c},b_{o}$—gate bias terms; ⊙—element-wise multiplication; σ(⋅) = sigmoid, tanh(⋅) are activation functions.

Functional interpretation: In the specific context of fatigue assessment, the gating mechanisms are tailored to capture cumulative risks. The forget gate ($f_{t}$) retains historical stability during low-risk intervals (output ≈ 1) but suppresses outdated low-risk states when sudden risk spikes occur, preventing underestimation. The input gate ($i_{t}$) amplifies updates during abrupt risk shifts, enabling the cell state ($c_{t}$) to accumulate rising risk trends promptly. Finally, given that fatigue correlates strongly with recent exertion, the output gate ($o_{t}$) enhances sensitivity as t approaches the window limit T, ensuring the hidden state $h_{t}$ reflects the worker’s immediate physiological status.

#### 3.3.3. Concatenate

The CNN branch produces global spatial features. These features are denoted as $Y_{gap}\in R^{128}$, obtained after the ECAConvBlock and the global average pooling layer. The LSTM branch generates global temporal features, denoted as $H_{2}\in R^{128}$, after processing sequential risk features across T time steps.

To integrate the spatial and temporal characteristics into a unified representation, the two feature vectors are fused by concatenation along the feature dimension, rather than by element-wise addition. This operation preserves the complementary information contained in each branch and results in a combined feature vector with doubled dimensionality. The fusion operation is expressed as follows:

(14) $$Y_{comb}=\left[Y_{gap};H_{2}\right]=Concat\left(Y_{gap},H_{2}\right)$$

This fused feature vector jointly encodes two types of key information:

(1) Local spatial associations extracted by the CNN branch;

(2) Temporal dependencies captured by the LSTM branch.

This full-feature foundation supports accurate predictions in later stages. After concatenation, the fused vector is passed through the two fully connected layers in the sequence. These layers reduce the data dimensionality while capturing the complex combination patterns of the merged features. To prevent model overfitting, a dropout mechanism is applied after each fully connected layer. The model employs the Adam adaptive gradient estimation optimizer, which combines the advantages of momentum gradient descent and adaptive learning rates. The final model outputs the following:

(15) $$\hat{y}=W_{out}\cdot Y_{comb}+b_{out}$$

where $\hat{y}$ represents the predicted fatigue index, which serves as a regressed approximation of the expert consensus score (based on the Borg RPE scale defined in Section 4.1.2); $W_{out}\in R^{1\times 256}$ denotes the output weights; $b_{out}$ represents the output bias. Distinct from traditional approaches that linearly map risk scores (e.g., simply summing RULA scores), this design enables the model to learn the complex, nonlinear mapping relationship between objective spatial–temporal posture features and the experts’ comprehensive fatigue assessment.

## 4. Experiments and Results

### 4.1. Experimental Settings

#### 4.1.1. Details

To verify the effectiveness and generalizability of the proposed method, this study selected 32 female workers (age 42 ± 5 years, height 1.60 ± 0.05 m, weight 51 ± 2.7 kg, all with three or more years of relevant work experience) from the manual edge-banding workstation for irregularly shaped furniture on an actual furniture production line of Onlead Office Furniture System Co, Ltd. (Nanjing, China). This ensured the subjects’ operational standardization and representativeness. Experimental equipment comprised a computer equipped with an AMD Ryzen 5 5600X 6-Core Processor (3.70 GHz CPU), an NVIDIA GeForce RTX 3070 Ti GPU, and the Windows 11 operating system to execute algorithmic code. Smartphones with fixed shooting angles of 45° or 135° from the side were used for data collection. The whole experiment was carried out in strict accordance with ethical rules. All participants provided informed consent before joining the study. To avoid interference from time-related confounding factors, the experiment was finished in three days. It was conducted from 9:00 a.m. to 12:00 a.m. each day.

#### 4.1.2. Dataset

This study used a smartphone camera to record a worker’s repeated manual edge-banding tasks. The recording covered eight work steps. It lasted about 30 min. Each step took 3 to 5 min. It is worth noting that these tasks matched the repeated operations in the workers’ daily shifts. This ensured the study mirrored real work situations. Later, the team used OpenCV to pick out key frames. These frames were preprocessed to the same size.

Data labeling (ground truth generation): The study aimed to create reliable fatigue labels for the dataset. It avoided wrong logic from making labels only from input features. So, three ergonomic experts were invited to mark the video segments. Their details are shown in Section 4.4.2.

(1) Scoring standard: The experts used the Borg Rating of Perceived Exertion (RPE) scale (6–20). They gave quantitative scores for the workers’ fatigue indexes. The scores were assigned to each divided work step.

(2) Assessment criteria: The experts were told to consider full visual signs. These signs were not limited to static joint angles. They included movement speed changes and compensatory postures. Shrugging and leaning are two such examples. They also considered the frequency of tiny pauses. All these signs show physical tiredness.

(3) Consensus: The final label $y_{i}$ for each sample was calculated as the average of the three experts’ scores to mitigate subjective bias. The inter-rater reliability among experts was validated (ICC > 0.85), ensuring the reliability of the ground truth.

**Figure 9:**
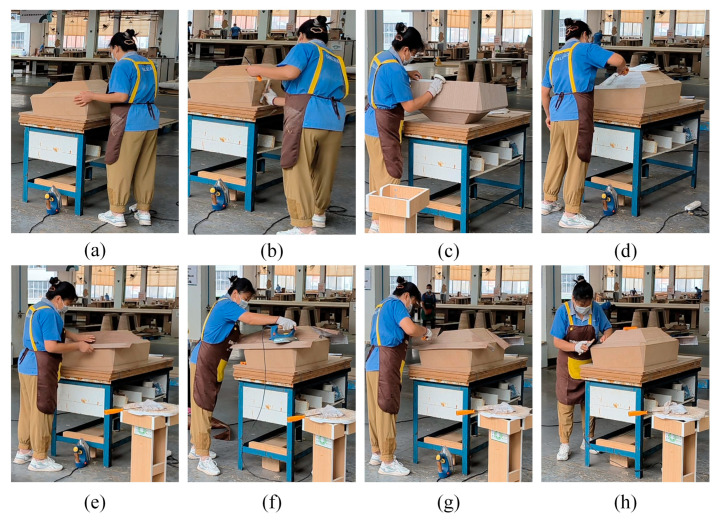
Covering eight workflows and arranged in sequence. (**a**) Preparation; (**b**) trimming; (**c**) tape application; (**d**) glue application; (**e**) veneer edge-banding; (**f**) ironing and heating; (**g**) trimming excess material; (**h**) finishing.

Data were divided into training, testing, and validation sets in a 7:2:1 ratio. To avoid temporal leakage, samples were randomly assigned to splits at the segment level rather than frame-contiguous blocks. Specific postures are illustrated in Figure 9.

#### 4.1.3. Evaluation Indicators

This study selects the mean squared error (MSE), mean absolute error (MAE), and coefficient of determination (R^2^) as metrics for evaluating model performance. Additionally, the model’s inference time is recorded to assess its real-time performance. The calculation formulas for each metric are as follows:

Loss function: mean squared error (MSE) is suitable for regression tasks, measuring the squared difference between predicted and actual values. A smaller value indicates higher accuracy. The formula is as follows:

(16) $$L_{MSE}=\frac{1}{N}\sum_{i=1}^{N}{\left(y_{i}-{\hat{y}}_{i}\right)}^{2}$$

where $y_{i}$ denotes the true fatigue index of the *i*-th sample, ${\hat{y}}_{i}$ represents the predicted value, and N is the number of samples.

Evaluation metric: mean absolute error (MAE) provides an intuitive measure of the average prediction error. A smaller value indicates lower prediction deviation in the model. The formula is as follows:

(17) $$MAE=\frac{1}{N}\sum_{i=1}^{N}|y_{i}-{\hat{y}}_{i}|$$

The coefficient of determination is used to measure a model’s ability to explain variations in actual data. The closer R^2^ is to 1, the stronger the model’s explanatory power. The formula is as follows:

(18) $$R^{2}=1-\frac{\sum_{i=1}^{N}(y_{i}-{\hat{y}}_{i}{)}^{2}}{\sum_{i=1}^{N}(y_{i}-\bar{y}{)}^{2}}$$

where $\bar{y}$ represents the mean of the true values.

### 4.2. Comparative Experiment

Multi-scenario posture monitoring data were integrated to extract nine core features. With the optimal model parameters derived from the training set as the baseline, this study compared the performance of three deep learning architectures: CNN, LSTM, CNN–LSTM, and the ECAConv–LSTM hybrid model. The experimental parameters were uniformly configured as follows: window size of 10, batch size of 16, 50 training epochs, and an Adam optimizer with a learning rate of 0.001. The comparison results are shown in Table 2. The results indicate that the ECAConv–LSTM hybrid model outperforms the others across all metrics, achieving an MSE as low as 0.028 and an R^2^ of 0.941. Regarding computational efficiency, the inference times recorded in Table 2 represent the post-processing phase (fatigue prediction based on extracted joint features). Although the introduction of the ECA mechanism increases the per-frame inference time to 0.71 ms (compared to 0.11 ms for the standalone LSTM), this computational cost is negligible. Standard monitoring cameras record video at 30 FPS. The time between two frames is about 33.3 ms. The module’s processing time is only 0.71 ms. It takes up less than 3% of the available time per frame. This result proves one key point. The proposed fatigue assessment module is very lightweight. It adds almost no extra delay to the MediaPipe pose estimation pipeline. This ensures smooth and real-time operation.

Crucially, the high coefficient of determination (R^2^ = 0.941) signifies a strong correlation between the model’s predictions and the expert consensus RPE scores. This demonstrates that the hybrid model has successfully learned to approximate the experts’ comprehensive evaluation of fatigue, rather than merely fitting a mathematical formula, while also validating the effectiveness of integrating spatial and temporal features.

In this section, the proposed hybrid ECAConv–LSTM model is compared with the models proposed in several recent studies. Table 3 presents a detailed comparison between these prior works and our method. As noted earlier, our hybrid ECAConv–LSTM model achieved the optimal performance in this study (R^2^ = 0.941). Compared to the static DNN model used by Md. Shakhaout Hossain’s [64] team, who processed 3D joint data for REBA risk level prediction (Accuracy = 89.07%), our hybrid model captures cumulative risk trends within time windows via its LSTM branch, achieving a 5.03 percentage point improvement in dynamic task monitoring. This result proves the key role of temporal features in fatigue prediction. Single DNNs find it hard to capture these features across different frames. Ereena et al. [30] used MediaPipe to get 2D joint points. They put these points into a traditional LSTM model. The model reached an R^2^ value of 0.9375. It was used for fatigue prediction in manual handling workstations. Our method shows better performance. Its result is similar to that obtained in the mentioned study but still stands out. This difference probably comes from the input feature choice made by these authors: they only used joint coordinates as input, and they did not include risk assessment factors like RULA, REBA, and OWAS. This shortcoming weakens the model’s ability: it cannot explain the mapping relationship between “posture risk and fatigue” clearly. Compared to single-criterion evaluations [55,69,70], this study integrates RULA /REBA/OWAS criteria, which are better suited for the complex risk scenario of “frequent upper limb movements” in manual edge-banding workstations. In their study, the same three assessment criteria were applied within a novel ergonomic risk analysis framework for 3D human posture estimation. Using the YOLOv3 model for triple risk [56] prediction achieved a maximum accuracy of 92%. However, the hybrid ECAConv–LSTM model employed in this paper outperforms YOLOv3. This superiority likely stems from our model’s integration of temporal and spatial feature hierarchies.

### 4.3. Ablation Experiment

**Figure 10:**
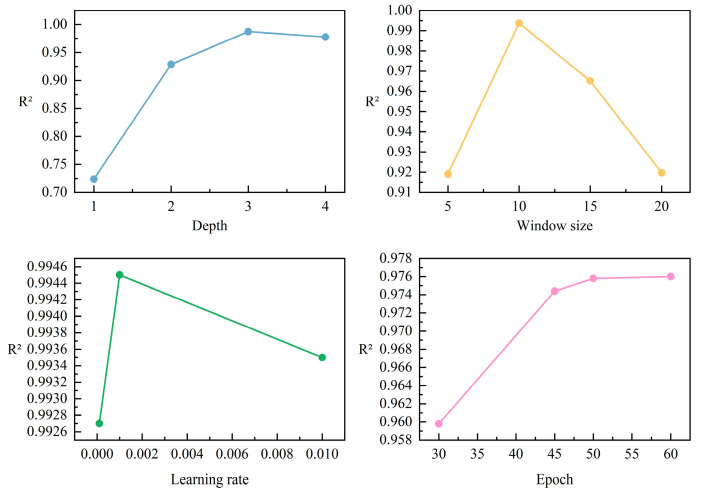
Parameter adjustment results.

To optimize model performance, the ECAConv–LSTM hybrid model was adopted as the baseline. This baseline model featured a configuration of two convolutional layers, two LSTM layers, and MaxPool layers. The experimental parameters were uniformly set as follows: temporal window size of 5, batch size of 16, 50 training epochs, and an Adam optimizer with a learning rate of 0.001. We conducted a series of experiments using the method of controlling variables to evaluate the impact of different parameters on model performance. In our study, the test progressively improved from 0.9232 to a significant 0.9945. Results are shown in Figure 10 and Table 4. Regarding pooling layer types, average pooling achieved optimal performance, likely because max pooling discards some important features. Epoch refers to the number of training iterations over the dataset. Larger epochs may lead to excessive training time, while smaller counts may result in suboptimal model performance. Experimental observations showed that, although the 60-epoch model achieved 0.976 performance, this represented only a 0.02% improvement over the 50-epoch model, accompanied by markedly longer training time. Thus, 50 epochs were selected for the subsequent experiments. For network depth, we set the LSTM branch to one–four layers while keeping the CNN branch (two layers of Conv1D) and fully connected layers (two layers of Dense) unchanged. We observed that a one-layer LSTM network showed significantly reduced performance, while a three-layer network yielded an optimal R^2^ (0.9874). This may be attributed to overfitting when the number of layers is excessive. A temporal window size that is too small risks losing critical temporal information, while an overly large size increases data redundancy. In this study, experiments were conducted with window sizes of 5, 10, 15, and 25 under the baseline model configuration, revealing that model performance was clearly optimal at a window size of 10. Different learning rates affect both the training phase and overall model performance. To determine the optimal learning rate, experiments were conducted with three distinct rates. The previously adopted learning rate of 0.001 maintained a test performance of 0.9945.

**Figure 11:**
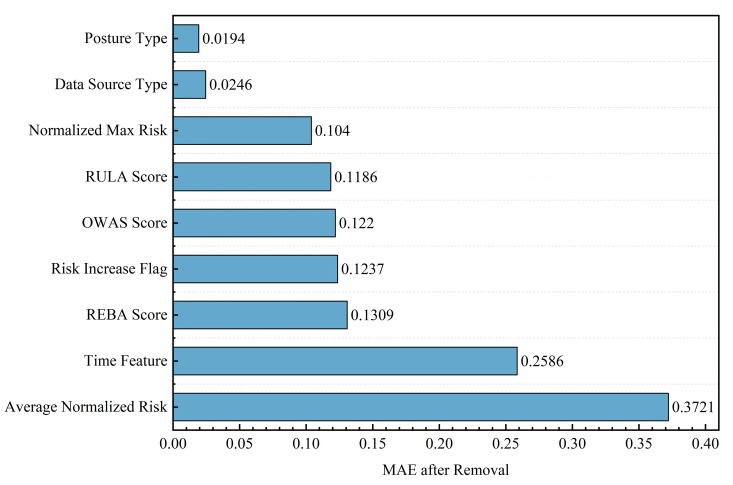
Feature ablation experiment results.

Under the optimal parameters, we performed ablation experiments on nine features using the “single-feature zeroing method,” setting all sample values of a certain feature category to 0 to simulate missing data. Feature importance was quantified via “MAE increment” (with higher increments indicating greater feature criticality). Detailed results are presented in Figure 11.

We analyzed the model from two aspects: performance and core elements. The ablation experiments we did show a clear result. “Average Normalized Risk” is the key feature for the model. Removing this feature leads to a sharp rise in the model’s MAE. The value jumps from around 0.103 to 0.3721. This fact confirms its top rank in feature importance. The next important feature is the “REBA Score”. Removing it causes the MAE to increase by 0.131. It plays a vital part in making sure the fatigue risk prediction works well.

While the “Average Normalized Risk” feature shows the highest importance, this does not imply that the model merely performs linear temporal smoothing. A simple smoothing algorithm (e.g., Moving Average) treats a “sustained static high-risk posture” and “high-frequency fluctuating movements” identically if their average scores are the same. However, physiologically, sustained static loads cause faster fatigue accumulation. The fact that the instantaneous “REBA Score” remains the second most critical feature (MAE increment of 0.131) is crucial evidence. It confirms that the model relies on the LSTM memory cells to capture the specific temporal dynamics and nonlinear accumulation effects from the instantaneous data sequence, rather than solely relying on the statistical mean. Thus, the rule-based features serve as a “strong anchor,” while the deep learning architecture refines the prediction based on temporal evolution.

To translate these statistical metrics into ergonomic decision-making utility, we consider the structure of the Borg RPE scale (range 6–20). In clinical practice, the minimum clinically important difference (MCID) for RPE is typically estimated at 1 to 2 units. Our model achieves an MAE of ≈0.10, which corresponds to less than 1% of the full scale and is an order of magnitude smaller than the resolution of human assessment (1.0 unit). This implies that the model’s prediction error is negligible for practical purposes, as it never deviates enough to shift the fatigue assessment across decision boundaries (e.g., misclassifying “Somewhat Hard” as “Hard”).

### 4.4. Real-World Scenario Results

#### 4.4.1. MediaPipe Pose Extraction Results

In this experiment, we mainly used three ergonomic assessment tools. These tools are RULA, REBA, and OWAS. They were applied to calculate ergonomic scores for worker postures. We combined the tool results with joint angle data. We carried out a quantitative analysis of human posture risks.

**Figure 12:**
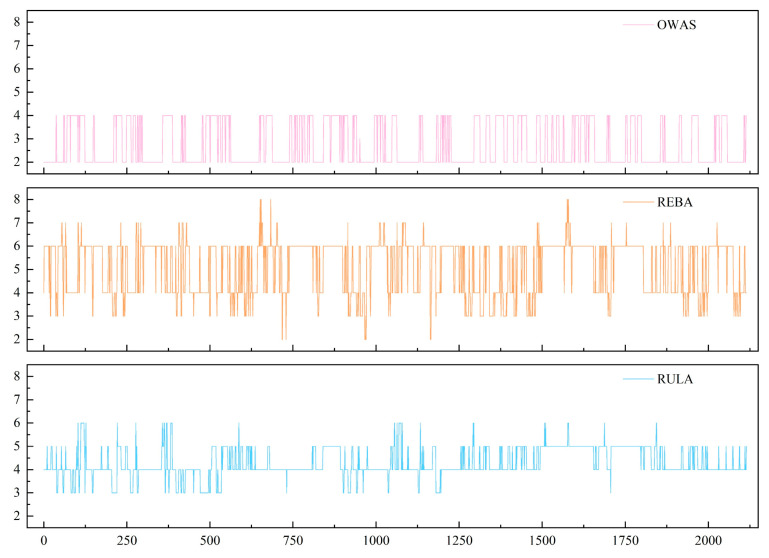
OWAS, REBA, and RULA scoring results.

Based on joint angle and coordinate data, the RULA, REBA, and OWAS scores were calculated. The results are shown in Figure 12.

The RULA scores were mostly 4 points (60.28%), followed by 5 points (30.21%). Scores of 3 points (7.23%) and 6 points (2.27%) were less common.

For REBA, scores concentrated at 6 points (55.13%), with lower frequencies at 4 points (32.58%), 3 points (8.56%), and 7 points (2.93%).

OWAS scores were mainly 2 points (64.54%), followed by 4 points (35.41%); no samples received a score of 1 point.

These distributions align with the operational nature of manual edge-banding stations, which involve “low load (≤10 kg) but frequent bending and twisting.”

Statistical analysis reveals two key observations: first, whole-body posture risk varies considerably during the workflow and may include extreme postures leading to high-risk scores; second, upper-limb posture risk remains relatively stable. Overall, postural risk is moderate in severity, though certain high-risk situations do occur. In addition, risk levels rise gradually as work time increases.

**Figure 13:**
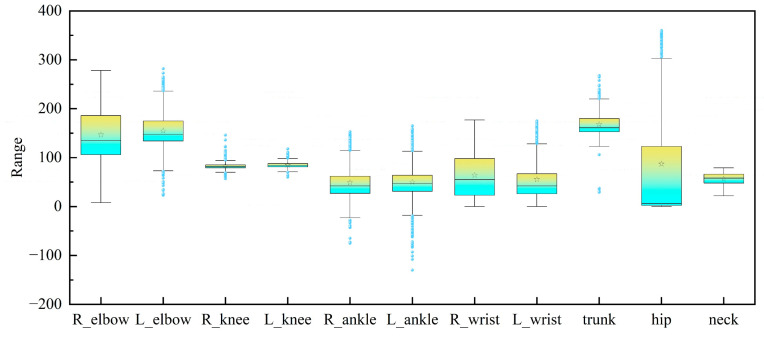
Angle range box plot.

Statistics from Figure 13 on key joint angles show clear patterns. Trunk angles are mostly between 140° and 200°. This range matches the worker’s typical bent-over working posture. Hip angles have the widest variation (0–358°). Angles of 0–10° (60.76%) mean the worker is standing upright. Angles of 120–358° (27.7%) mean the worker is in twisting postures. The large range of angles during bending and twisting shows that this diversity needs focused attention. Right elbow and left elbow angles have big differences. This shows that workers have a habit of using only one side of their body during their work.

The box plots demonstrate that the angle ranges of both the right and left knees are narrow, indicating relatively low knee flexibility and limited variability in joint angles. Box plots of symmetrical body parts look similar but have small differences. This shows the body has posture balance, but there may be potential asymmetry problems. Box plots for upper and lower limbs show a clear difference. Upper limbs have greater movement flexibility. Their angle variation range and dispersion are distinctly different from those of lower limbs.

#### 4.4.2. Expert Assessment of Consistency Validation

We invited three ergonomic assessment experts. Each of them has more than five years of work experience. They scored the video frames on their own. We aimed to make the validation process comprehensive. So, the experts carried out two different types of assessments:

Objective scoring: the experts used RULA, REBA, and OWAS scales to determine whether the system’s input features are accurate.

Subjective fatigue labeling: This involved assigning a fatigue index (based on the Borg RPE scale) to the video segments to serve as the ground truth for model training.

Intraclass correlation coefficients (ICCs) and Cohen’s kappa coefficients were employed to validate the consistency between system scores and expert evaluations, as well as the inter-rater reliability among experts for the fatigue labels.

In the expert reliability assessment, the ICC(3,1) model, as a single-measurement form of the two-way mixed-effects model, can be applied in research scenarios where the raters are fixed effects. Its mathematical expression is as follows:

(19) $$ICC(3,1)=\frac{{MS}_{rater}-{MS}_{error}}{{MS}_{rater}+(k-1){MS}_{error}+\frac{k}{n}({MS}_{subject}-{MS}_{error})}$$

Among these, ${MS}_{rater}$ denotes the mean square between raters, ${MS}_{subject}$ denotes the mean square between subjects, ${MS}_{error}$ denotes the residual mean square, k = 3 represents the number of raters, and *n* denotes the number of subjects.

For method validation, the ICC(*A*,1) model, as a one-way random effects model, is specifically designed to assess the consistency between a new measurement method and a benchmark. This study adopts the average of three experts’ ratings as the benchmark, calculates the covariance between the scores from the proposed method and the expert average, and derives the reliability estimate through the following approach. Its mathematical expression is as follows:

(20) $$ICC(A,1)=\frac{\sigma_{between}^{2}}{\sigma_{between}^{2}+\sigma_{within}^{2}}$$

Among these, $\sigma_{between}^{2}$ represents the between-group variance (systematic variation between expert means and the method proposed in this paper), while $\sigma_{within}^{2}$ denotes the within-group variance (individual measurement error).

The ICC(3,1) values of expert ratings are shown in Table 5. These values are all higher than 0.8. They prove that the experts’ assessments have good consistency. This result supports a key step. We can use the average of expert scores as the benchmark value. It is used for ICC(*A*,1) analysis. The proposed evaluation method also shows good consistency. It matches the expert benchmarks well. The consistency covers RULA, REBA, and OWAS scores. All comparison results have significance levels below 0.001. Their ICC(*A*,1) values are all higher than 0.75. It is worth noting that the ICC(A,1) value for the RULA total score reaches 0.906. Values above 0.90 reflect excellent reliability. This confirms that the method has good discriminant validity. It is comparable to the expert panel in comprehensive risk assessment.

Crucially, for the fatigue index (ground truth) based on the Borg scale, the experts also demonstrated high agreement (ICC(*A*,1) = 0.892), validating the use of the averaged expert score as the reliable ground truth for model training.

Cohen’s kappa coefficient is further used to assess consistency. As a statistical measure commonly employed to evaluate agreement in categorical data, this coefficient is typically applied to quantify inter-rater consistency (agreement between different raters). The coefficient ranges from −1 to 1, with higher values indicating stronger agreement. Specifically, a value of 1 denotes perfect agreement, 0 indicates agreement equivalent to random chance, and values below 0 signify agreement worse than random chance.

(21) $$k=\frac{p_{o}-p_{e}}{1-p_{e}}$$

where $p_{o}$ denotes the actual agreement ratio, while $p_{e}$ represents the expected agreement ratio (under random conditions).

Table 6 presents the Cohen’s kappa results for the comparison between the proposed method and expert ratings. For this analysis, the scores from the three experts are first averaged to form the expert benchmark, and the kappa coefficient is then calculated based on this benchmark and the proposed method’s scores. Both RULA and OWAS yield kappa values exceeding 0.75 (“Good consistency”), indicating good consistency between the proposed method and expert benchmarks. REBA yields a value of 0.710 (“substantially consistent”). Although the value does not go above 0.75, it is still acceptable. It shows that the assessment matches expert opinions on REBA scores well. Only small differences exist between them. In general, the proposed method has good consistency. It meets the evaluation needs of industrial scenarios. It provides high reliability for the final judgment of risk levels.

#### 4.4.3. Model Prediction Results

The final predicted risk scores are mapped to four fatigue indexes, and the model outputs are presented in Table 7. A human fatigue risk analysis of the sequential work steps (a–h) identifies three low-risk steps: b, d, and e. During these steps, workers exhibit low fatigue indexes, and their work methods are deemed acceptable. Steps a, c, f, and g are classified as medium-risk steps. Workers in these steps exhibit noticeable fatigue, necessitating mitigation measures such as scheduling short breaks every 30–45 min and optimizing ergonomic workstation design. Step h has a predicted fatigue index of 3.12 (on the normalized scale mapping to high RPE), which is mapped to the “high-risk” category under the four-tier fatigue classification. Given the cumulative fatigue accumulation across eight steps, in addition to implementing measures for medium-risk steps, the following measures are further recommended: (1) implement task rotation every 1–2 h; (2) provide ergonomic training for workers; and (3) deploy mechanical aids to assist with repetitive tasks. Assessment results suggest workers feel fatigued during high-risk jobs. Working in the same position for long periods causes fatigue, and this weariness can strain muscles, leading to multiple issues. Unfocused workers may perform less effectively and precisely. Also, safety issues may arise. The system strongly recommends rotating the worker’s tasks immediately to fix this, keeping tiredness at bay.

To rigorously evaluate the real-time capability of the deployed system, we measured the end-to-end latency per frame. This includes two sequential stages: (1) upstream pose estimation using MediaPipe; (2) downstream fatigue prediction with our proposed ECAConv–LSTM model. Tests were conducted on our hardware platform (Ryzen 5 5600X CPU). The results show that the actual total system latency is about 21 ms per frame. This equals a system-level throughput of around 47 FPS. This performance is much higher than the standard 30 FPS industrial video capture rate. It proves that the integrated system supports smooth real-time monitoring. There is no frame accumulation or lag.

In terms of the overall process, the existing tasks could lead to cumulative fatigue. For steps assessed as medium risk, priority should be given to measures such as scheduled short breaks and ergonomic workstation adjustments.

Meanwhile, some measures need urgent application in the whole process. These include job rotation, training, and mechanical assistance. They can reduce safety or efficiency risks caused by fatigue. For low-risk steps, we need to monitor workers’ postures and operations continuously. In this way, workers will not become too tired during the whole operation.

## 5. Discussion

### 5.1. Interpretation of Feature Importance and Fatigue Mechanisms

To verify the contribution of each selected feature, we conducted a comprehensive ablation study (detailed in Section 4.3 and Figure 11). The rigorous analysis of feature importance reveals the underlying mechanisms of fatigue generation:

(1) Dominance of cumulative metrics: The “Average Normalized Risk” emerged as the most critical determinant, with its removal causing the largest spike in mean absolute error (MAE increment of 0.3721). This empirically confirms that fatigue is a time-integral process. A high instantaneous risk score does not necessarily imply fatigue, but a sustained high average risk over the time window (T = 10) is a definitive predictor.

(2) Validity of expert rules: The “REBA Score” rank second in importance (MAE increment of 0.131). This indicates that the global body posture assessment provided by REBA is more informative for fatigue prediction than local joint angles alone. It validates our “dual-drive” hypothesis that deep learning models benefit significantly from the guidance of structured ergonomic knowledge.

(3) Robustness of contextual features: While “data source type” and “perspective encoding” showed lower impact on MAE (0.0246), their inclusion remains methodologically justified. These features act as stabilizers, preventing the model from learning erroneous correlations due to camera angle biases. Although removing them has a minor impact on the test set (which has similar distributions), they are essential for the system’s potential generalization to new camera setups in future deployments.

### 5.2. Comparison with State-of-the-Art Methods

The comparative analysis shows that our proposed “dual-drive” framework (R^2^ = 0.941) statistically outperforms existing methods. This performance aligns with broader trends in deep learning, where specialized architectures have solved complex pattern recognition tasks in diverse fields. For instance, advanced sequence learning approaches have proven highly effective in decoding temporal dynamics for emotion recognition [71], while robust image classification models have demonstrated efficacy in extracting features from deteriorated visual data, such as historical manuscripts [72]. Drawing inspiration from these cross-domain successes in sequential modeling and visual feature extraction, our framework adapts these paradigms to industrial fatigue assessment, quantifying its advantages in three key aspects:

(1) Dynamic vs. static assessment: Hossain et al. [55] used a static DNN for REBA prediction. They achieved an accuracy of 89.07%. In contrast, our dynamic model captures cumulative fatigue, resulting in a superior fit (R^2^ = 0.941, explaining >94% of variance). Internally, compared to the static CNN baseline (Table 2), our hybrid approach reduces the mean squared error (MSE) by 56.9% (from 0.065 to 0.028). This performance difference proves a key point. Static models cannot capture cumulative fatigue from sustained holding postures. This factor is critical in edge-banding tasks. Our LSTM branch models it successfully.

(2) Knowledge-guided vs. pure-data-driven approach: Bagga et al. [30] used a similar LSTM architecture; they only relied on raw joint coordinates and their model achieved an R^2^ of 0.9375. However, our “Dual-Drive” framework, which embeds expert rules (RULA/REBA scores) into the feature space, outperforms this pure-data-driven baseline. As shown in our ablation study (Table 2), enhancing a standard LSTM with these rule-based features and the ECA mechanism reduces the prediction error (MSE) by 44% (from 0.050 to 0.028). It helps the model tell the difference between “biomechanically awkward” and “safe” postures. It works even when coordinate changes are small. In this way, the model explains the “posture risk–fatigue” mapping relationship effectively.

(3) Attention-enhanced vs. general purpose: Paudel et al. [56] used the generic YOLOv3 model for ergonomic risk analysis. They achieved an accuracy of 92%. In contrast, our specialized ECAConv network utilizes channel attention to focus specifically on task-relevant body parts. Furthermore, our proposed context-aware adaptive pooling strategy formally addresses the varied nature of industrial actions. Unlike general-purpose object detectors that often smooth out transient signals, our adaptive mechanism (splitting features at T = 7) preserves high-frequency risk signals from rapid movements, ensuring that momentary but dangerous deviations are not lost in the temporal averaging.

### 5.3. Robustness Analysis: Impact of Pose Estimation Errors

The use of pseudo-3D data from MediaPipe inherently contains metric errors compared to gold-standard motion capture systems. However, our analysis suggests that the proposed system possesses a high tolerance for such deviations due to three key factors:

(1) The “Buffering Effect” of Discrete Scoring: Ergonomic assessment tools like RULA and REBA function as step functions, converting continuous joint angles into ordinal scores (e.g., a trunk flexion of 10° and 18° both yield a score of 1). This discretization acts as a natural buffer. Unless the estimation error crosses a specific critical threshold (e.g., the 20° boundary), the final risk score remains unaffected.

(2) Temporal smoothing via LSTM: Random jitters in frame-level pose estimation are further mitigated by the proposed LSTM network. By aggregating temporal features over a window (T = 10), the model focuses on the trend of posture rather than instantaneous noise, filtering out high-frequency outliers caused by estimation instability.

(3) Empirical validation: Most importantly, the high consistency between the system’s output and manual expert ratings (ICC > 0.88 for fatigue index, as shown in Table 5) serves as empirical proof. Since experts scored based on visual ground truth, the strong correlation indicates that the intermediate pose estimation errors were statistically insufficient to decouple the system’s judgment from the true ergonomic reality.

### 5.4. Practical Implications for Smart Manufacturing

Besides algorithm performance, this study provides practical guidance for occupational health management in labor-intensive industries.

(1) From “passive monitoring” to “process re-engineering”: Step ‘h’ (finishing) is identified as a high-risk operation. Its predicted fatigue index reaches 3.12. This result points to a structural problem in the workflow. Managers should not just blame workers for bad postures. Instead, they should focus on process re-engineering. For example, they can introduce height-adjustable workstations. They can also use mechanical aids specially for the finishing stage. These measures can stop fatigue from building up.

(2) Fatigue heatmaps: Wearable sensors like IMUs or sEMG may affect precise operations. The proposed system is a cheap and non-invasive alternative. It can be connected to existing factory CCTV networks. It can generate real-time “fatigue heatmaps” for the production line. This data-driven method helps safety managers change their work mode. They can shift from analyzing accidents after they happen to advanced risk prevention.

### 5.5. Limitations and Future Directions

While the proposed system demonstrates robust performance, several limitations warrant further investigation to enhance its generalizability and practical applicability.

(1) Dataset bias and external validity: The current dataset is limited to 32 female workers, introducing a specific selection bias. This demographic choice reflects the operational reality of the manual edge-banding workstations in the partner facility, where female workers constitute the vast majority of the staff, thus ensuring high ecological validity for this specific industrial scenario. However, this limits the external validity of the trained model parameters when applied to male populations. Due to sexual dimorphism in anthropometry (e.g., limb length ratios, center of gravity) and physiological fatigue thresholds (e.g., upper body muscle strength), the model may require transfer learning or parameter re-calibration for male-dominated workstations. Nevertheless, the proposed “dual-drive” methodological framework—integrating biomechanical rules with temporal deep learning—is generic and can be adapted to diverse demographics provided that representative training data are available. Future studies will expand the dataset diversity to include male subjects and a wider age range to test cross-population robustness.

(2) Occlusion in complex environments: The system currently relies on monocular pose estimation. Although the experimental setup used controlled angles, heavy occlusion remains a challenge in cluttered, unconstrained industrial environments. Standard monocular algorithms may fail to infer joint positions when body parts are blocked by machinery or workpieces. Future iterations will explore multi-view fusion algorithms and temporal in-painting techniques. By leveraging historical trajectory data to reconstruct occluded joints, we aim to enhance the system’s resilience to visual obstructions.

(3) Edge deployment optimization: Finally, while the current inference speed supports near real-time analysis on a desktop GPU, deployment on edge devices with limited computing power requires further efficiency. The model currently operates without compression. Future work will focus on model quantization and lightweight architecture variants (e.g., pruning redundant channels in the ECA module) to reduce the computational footprint, enabling direct integration into low-cost embedded monitoring systems on the factory floor.

(4) Comparative constraints of model architectures: Finally, the experimental comparison in this study was primarily conducted against standard CNN and LSTM baselines to validate the efficacy of the proposed modules. We did not extensively benchmark against recent heavyweight architectures (e.g., Vision Transformers or Spatio-Temporal Graph Convolutional Networks) or pure end-to-end approaches that completely bypass ergonomic rule inputs. This decision was driven by the strict constraint of low-latency edge deployment, which many complex SOTA models struggle to meet. Future work will conduct a broader architectural comparison to rigorously quantify the trade-offs between the accuracy gains of these advanced deep learning paradigms and the computational efficiency required for factory integration.

(5) Validation of intervention efficacy: While the system provides actionable recommendations (e.g., job rotation triggers based on the fatigue index), the actual efficacy of these interventions in reducing long-term WMSD incidence remains conceptually proposed rather than empirically validated within the scope of this study. The current research focuses on the accuracy of the “diagnostic tool” (the assessment system). Future work will involve longitudinal field studies to implement these feedback loops on the shop floor and statistically measure the “before-and-after” impact on worker health indicators, thereby closing the loop between detection and prevention.

(6) Subject-dependent validation constraints: As described in Section 4.1.2, the dataset was split randomly at the segment level. While this strategy successfully prevents frame-level temporal leakage, we acknowledge that segments from the same subject may appear in both training and test sets. Given the dataset size (N = 32), this subject-dependent evaluation was chosen to ensure stable model convergence by maximizing feature variance during training. We recognize that this may limit the strict assessment of generalization to unseen users. Future work will employ Leave-One-Subject-Out (LOSO) cross-validation on an expanded dataset to rigorously verify subject-independent performance.

(7) Monocular vision constraints: While MediaPipe provides reliable pose estimation, angles are primarily derived from 2D projections with pseudo-3D depth estimation. We acknowledge that monocular depth ambiguity and perspective distortion (relative to the vertical axis) introduce geometric errors compared to multi-camera motion capture systems. To mitigate this, we explicitly included the Perspective ID ($P_{id}$) as an input feature (Table 3), enabling the model to learn latent compensation logic for different camera viewpoints. However, precise quantification of this angular error requires ground-truth validation (e.g., Vicon systems), which is reserved for future calibration studies.

## 6. Conclusions

This study creates a three-stage prevention system for WMSDs. It targets manual edge-banding workstations in furniture factories. The system makes breakthroughs in both performance and practical use. Specifically, the system uses the lightweight, real-time MediaPipe pose estimation framework. It also applies vector geometry algorithms to calculate core joint angles.

This allows the system to reliably extract workers’ key joint coordinates in real time and provides accurate, automated scoring based on three widely used ergonomic assessment standards: RULA, REBA, and OWAS. The evaluation results show agreement with expert assessments in both objective posture scoring and subjective fatigue ratings, confirming the system’s reliability. The consistency covers both objective posture scoring and subjective fatigue assessment. This proves the system is reliable. Ablation experiments further find critical parameters. “Average Normalized Risk” is the core feature for fatigue prediction. The system combines a window size of 10 and a 3-layer LSTM. This combination keeps critical risk features from the past 5–10 s. It also reduces interference from occasional low-risk postures with the LSTM’s forget gate. This design accurately captures short-term risks during workers’ fast movements. It improves both the timeliness and performance of risk detection. For fatigue prediction, the system uses an ECAConv–LSTM hybrid model. It improves performance by 9.4% compared with standalone CNN models. It also improves by 5.5% compared with standalone LSTM models. This hybrid model works better than individual single-modality models and existing related studies. However, the study has some limitations. The dataset does not include diverse types of workers. Complex on-site environments are not simulated. This may reduce the accuracy of joint extraction by MediaPipe. The model’s inference latency is still not good enough for real-time industrial alert systems. In the future, researchers will focus on several tasks. We will expand the sample size and add more demographic diversity. We will optimize algorithm efficiency and integrate multimodal data. We will also develop lightweight model variants to solve these problems.

In summary, the three-stage prevention system developed in this study can automatically and accurately assess and predict WMSD risks. It targets manual edge-banding workstations. It provides enterprises with practical WMSD prevention solutions. It supports the development of occupational health management systems. It also offers scientifically valid engineering references for furniture manufacturing and other labor-intensive industries.

## Figures and Tables

**Table 1:** Definitions of input features.

Feature Category	Symbol	Range/Type	Definition/Derivation Logic
Expert Scores	*X_RULA_*, *X_REBA_*, *X_OWAS_*	Int: [1, 7], [1, 15], [1, 4]	Instantaneous risk scores (corresponding to RULA Score, REBA Score, OWAS Score) derived from standard tables.
Avg. Norm. Risk	${\bar{R}}_{norm}$	Float: [0, 1]	Mean of min–max-normalized expert scores over window T: ${\bar{R}}_{norm}=\frac{1}{T}\sum_{1}^{T}Mean(Norm(X_{t}))$
Max. Norm. Risk	$R_{max}$	Float: [0, 1]	The maximum normalized risk value among the three expert scores in the current frame.
Risk Trend	$I_{trend}$	Binary: {0, 1}	Set to 1 if the aggregated risk score strictly increases for 3 consecutive frames; otherwise, 0.
Context Metadata	$P_{type}$ $,D_{src}$ $,T_{feat}$	Categorical/[0, 1]	Contextual metadata features: Posture Type ($P_{type}$): encoding for camera viewpoint (front/side). Data Source Type ($D_{src}$): ID for the capture device. Time Feature ($T_{feat}$): relative timestamp (t/T).

**Table 2:** Comparison of experimental results.

Model	MSE	MAE	R^2^	Time (ms)
CNN	0.065	0.147	0.860	0.23
LSTM	0.050	0.150	0.892	0.11
CNN–LSTM	0.033	0.135	0.924	0.56
ECAConv–LSTM	0.028	0.100	0.941	0.71

**Table 3:** Compared with other studies.

Author	Dataset	Model	Guidelines	Performance
Md. Shakhaout Hossain [55]	Human 3.6 m	DNN	REBA	Accuracy = 89.07%
JoonOh Seo [69]	Custom dataset	SVG	OWAS	Accuracy = 89%
Seong-oh Jeong [70]	Custom dataset	Mediapipe	REBA	-
Prabesh Paudel [56]	Human 3.6 m, COCO, MPII	YOLOv3	RULA, REBA, OWAS	Accuracy = 92%
Ereena Bagga [30]	Human 3.6 m	LSTM	-	0.9375
Our research	Custom dataset	ECAConv–LSTM	RULA, REBA, OWAS	0.941

**Table 4:** Changing the type of pooling layer.

Pooling Layer	Max	Average
R^2^	0.9232	0.9257

**Table 5:** Explanation of consistency between system scores and expert scores.

Guidelines	ICC(3,1)	Reliability Explanation	ICC(A,1)	Reliability Explanation	*p*
RULA	0.807	Good consistency	0.894	Good consistency	<0.001
REBA	0.862	Good consistency	0.886	Good consistency	<0.001
OWAS	0.879	Good consistency	0.754	Good consistency	<0.001
Fatigue Level (RPE)	0.885	Good consistency	0.892	Good consistency	<0.001

**Table 6:** Cohen’s kappa results for expert agreement with the scoring method in this paper.

Guidelines	Cohen’s Kappa	Consistency Strength	*p*
RULA	0.755	Good consistency	<0.001
REBA	0.710	Substantially consistent	<0.001
OWAS	0.768	Good consistency	<0.001

**Table 7:** Prediction results.

Steps	a	b	c	d	e	f	g	h
Pred. Fatigue Index	2.24	1.65	2.06	1.94	1.68	2.07	2.24	3.12
Fatigue index	Moderate Risk	Low Risk	Moderate Risk	Low Risk	Low Risk	Moderate Risk	Moderate Risk	High Risk

## Data Availability

The source code of this study is available upon request. However, the videos from furniture factories used in data collection are not publicly accessible, as this is necessary to protect the privacy and personal information of the workers involved in this research.

## Abbreviations

The following abbreviations are used in this manuscript:

WMSDs	work-related musculoskeletal disorders
MSDs	musculoskeletal disorders
ILO	International Labour Organization
REBA	Rapid Entire Body Assessment
RULA	Rapid Upper Limb Assessment
OWAS	Ovako Working Posture Analyzing System
EMG	electromyography
sEMG	Surface Electromyography
PAF	Part Affinity Fields
CNN	Convolutional Neural Networks
HRNet	High-Resolution Net
LSTM	Long Short-Term Memory
ST-GCN	Spatio-Temporal Graph Convolutional Network
MLP	Multilayer Perceptron
AlexNet	Deep Convolutional Neural Network
ResNet	Residual Network
SENet	Squeeze-and-Excitation Networks
CBAM	Convolutional Block Attention Module
DA	Dual Attention Network
ECA	Efficient Channel Attention
ADHD	attention deficit hyperactivity disorder
EEG	Electroencephalogram
NMQ	Nordic Musculoskeletal Questionnaire
IMU	inertial measurement units
EG	electrogoniometry
SVMs	Support Vector Machines
MSE	mean squared error
MAE	mean absolute error
R2	R-Square
DNN	Deep Neural Network
ICC	Intraclass correlation coefficients
RPE	Borg Rating of Perceived Exertion
